# Bacteriophages as Potential Sustainable Alternatives to Antibiotics for Controlling *Salmonella* in the Poultry Value Chain

**DOI:** 10.3390/antibiotics15060628

**Published:** 2026-06-22

**Authors:** David Yembilla Yamik, Kitiya Vongkamjan, Vincent Guyonnet, Warangkana Kitpipit, Wattana Pelyuntha

**Affiliations:** 1 Department of Biotechnology, Faculty of Agro-Industry, Kasetsart University, Chatuchak, Bangkok 10900, Thailand; davidyembilla.y@ku.th (D.Y.Y.); kitiyavongkamjan.a@ku.th (K.V.); 2 Département de Sciences Cliniques, Faculté de Médecine Vétérinaire, Université de Montréal, Saint-Hyacinthe, QC J2S 2MC, Canada; vincent.guyonnet18@gmail.com; 3 Akkhraratchakumari Veterinary College, Walailak University, Thasala, Nakhon Si Thammarat 80160, Thailand; warangkana.ki@wu.ac.th; 4 Futuristic Science Research Center, School of Science, Walailak University, Thasala, Nakhon Si Thammarat 80160, Thailand; 5 Research Center for Theoretical Simulation and Applied Research in Bioscience and Sensing, Walailak University, Thasala, Nakhon Si Thammarat 80160, Thailand

**Keywords:** *Salmonella*, bacteriophages, poultry value chain, antimicrobial resistance, One Health

## Abstract

*Salmonella* remains one of the most critical zoonotic pathogens in the poultry sector, linked to animal disease, foodborne illness, and the global crisis of antimicrobial resistance (AMR). Poultry acts as a major reservoir, enabling *Salmonella* transmission from hatchery to retail products through horizontal, vertical, and environmental routes. Despite the use of biosecurity, vaccination, antibiotics, and chemical decontamination, effective and sustainable control across the poultry value chain remains difficult, particularly in the face of rising multidrug-resistant strains and growing consumer concerns over chemical residues. Bacteriophages (phages), viruses that selectively infect and lyse bacteria, have emerged as a promising biological alternative for *Salmonella* control. Although many studies have reported the effectiveness of phages against bacterial species, including *Salmonella*, in the poultry industry, reports on their full potential to combat antimicrobial-resistant *Salmonella* across the entire poultry value chain remain limited. Therefore, this review synthesizes current evidence on the application of phages throughout the poultry value chain, including on-farm interventions, processing plant decontamination, and food packaging and storage. Findings from the reviewed articles indicate over a 90% reduction in *Salmonella* spp. in poultry farms and post-harvest meat, along with lower mortality in phage-treated groups compared to untreated groups; however, these outcomes depend on several factors (e.g., phage strains, concentrations, application methods, and environmental conditions). Laboratory, pilot, and field studies consistently demonstrate that phage preparations, especially when formulated as cocktails or combined with complementary interventions, can achieve substantial reductions in *Salmonella*, including antibiotic-resistant serovars, in live birds, eggs, poultry environments, and meat products. Unlike antibiotics and chemical sanitizers, phages act with high specificity, preserving beneficial microbiota and maintaining the sensory and nutritional quality of poultry products. Their safety has been supported by toxicological and genomic assessments, and several phage-based products have obtained regulatory approval, including Generally Recognized as Safe (GRAS) status for food applications in the United States. By integrating efficacy, safety, regulatory, and practical deployment data, this review highlights bacteriophages as a scientifically validated and One Health–aligned tool capable of reducing *Salmonella* transmission from farm to fork across the poultry value chain, thereby laying the foundation for their future adoption in the poultry industry. Phage-based interventions offer a sustainable pathway to enhance food safety, limit antimicrobial resistance (AMR) dissemination, and strengthen consumer confidence in poultry products. However, the major limitation is the emergence of phage-resistant bacterial strains, as well as the potential involvement of some phages in the transfer of resistance and virulence genes, which could raise public concern. Nevertheless, the use of phage cocktails and whole-genome sequencing, involving tools such as ResFinder and virulence finder, can facilitate the selection of safe phages for application.

## 1. Introduction

Poultry are domesticated birds (chickens, ducks, turkeys, geese, guinea fowl, etc.) that are reared primarily for meat and eggs. They represent a high-quality, environmentally sustainable food source (meat and eggs), contributing to global protein supply, nutritional adequacy, food security, and economic growth [1,2,3]. With the world population projected to exceed 9.2 billion by 2050 [4], the total global food demand is expected to increase by 35–56% [5], with a 73% increase in meat demand [6]. According to the Food and Agriculture Organization (FAO) [7], the poultry industry has emerged as a robust sector worldwide, growing by 32.7% between 2013 and 2023 to reach a global output of 138 million tons of poultry meat in 2023. The sector is greatly contributing to meet the rising global demand for animal protein. However, the intensification of poultry production and processing may raise concerns about the safeguarding of flocks against bacterial infections or contamination, with *Salmonella* spp. being the most commonly identified (poultry serving as reservoirs). *Salmonella* is one of the most significant foodborne pathogens in the poultry industry, causing both public health concerns and economic losses [8,9]. As the causative agents of salmonellosis in both poultry and humans, they are classified as zoonotic pathogens of public health significance [8,10]. Globally, the burden of salmonellosis is estimated to exceed 20 million cases annually, resulting in approximately 150 thousand deaths [8], thus highlighting the urgent need for novel and effective prevention and treatment strategies.

Traditionally, a wide range of approaches, including antibiotics, vaccines, hygiene practices, chemical agents, probiotics, prebiotics, and other food supplements, have been used by the poultry sector to control *Salmonella* spp. However, some of these measures may lead to undesirable outcomes, with consumers less likely to consume poultry products [11]. For instance, some chemical treatments may potentially leave the presence of residues in poultry products, affecting the overall safety of food and consumer acceptability [12,13,14]. Also, the cost and complexity of biosecurity measures may lead to non-compliance by farmers [15,16]. In addition, a lack of trained farmers may limit the use of phytobiotics, while inconsistent outcomes due to variations in efficiency, strain specificity, cost, and short-term protection may limit the use of probiotics and vaccines. These limitations, combined with increasing concerns over the development of antimicrobial resistance (AMR), present some significant challenges for the control of *Salmonella* by the poultry sector [17]. Although antibiotics are the frontline strategy to treat animal diseases, their misuse in animal production for the purpose of maximizing productivity has given rise to numerous antibiotic-resistant bacterial strains [17]. These resistant bacteria can be transmitted from farms to the environment and then eventually to humans, thus creating a public health emergency [17,18]. It is projected that deaths caused by antibiotic-resistant bacteria may reach 10 million globally by 2050 [19]. Additionally, the World Health Organization (WHO) predicts that multidrug-resistant bacteria will become the leading cause of death by 2050 [20]. As of 2024, the WHO has classified *Salmonella* as a high-priority research pathogen due to its resistance to antibiotics, particularly to fluoroquinolones [21].

Consequently, novel and effective alternatives are needed for the control of *Salmonella* spp. within the poultry value chain to safeguard food safety and maintain consumer confidence. The promising alternative is bacteriophage (phage) therapy, a biological control method that targets and kills specific bacterial pathogens without harming the beneficial microbiota. Phages are natural viruses that infect and replicate within target bacteria, killing them in the process. Phage activity has shown some promise against both Gram-negative and Gram-positive bacteria, exhibiting high specificity and precision [22,23]. Moreover, phage treatment has a minimal impact on the sensory and organoleptic properties of poultry products [24,25,26]. Studies have demonstrated the efficacy of phages against foodborne bacterial pathogens in chicken products [26,27], with significant reductions in poultry farms for various *Salmonella* serovars [23,28,29]. However, despite their efficacy, there are limited reports on the holistic application of phages for the control of *Salmonella* along the poultry value chain, from farm to fork.

Therefore, this review aims to explore the potential of phages as an innovative solution for controlling *Salmonella* in the poultry value chain. It will examine various applications and their effectiveness, as well as lay the foundation for developing effective phage-based interventions to ensure safer poultry products for consumers. In this review, the significance of foodborne bacterial pathogens, with priority given to *Salmonella* spp. in the poultry value chain, is discussed to explain the focus on *Salmonella*. This is followed by an overview of current traditional control methods and their limitations, providing justifications for considering phage treatment as an alternative. This review also examines phage-based control strategies from farm to fork, including regulatory and safety considerations as well as challenges and limitations of phage applications, acknowledging that although phage-based control is promising, certain factors may limit its full commercialization and adoption.

A systematic search of published studies on the use of phages to control *Salmonella* spp. in poultry was conducted using databases such as Google Scholar, PubMed, Scopus, Web of Science, and ScienceDirect with relevant keywords (e.g., chicken, *Salmonella*, phage; bacteriophage and *Salmonella* and poultry; phage therapy *Salmonella* chicken; *Salmonella* control broilers bacteriophage). Articles were selected based on their relevance to phage efficacy, experimental factors, and outcomes such as pathogen reduction, often excluding non-poultry or non-*Salmonella-*focused studies.

## 2. The Significance of Bacterial Pathogens in Food Safety

Foodborne pathogens comprise bacteria, viruses, and parasites that cause a wide range of illnesses [30,31,32]. Among these, several bacterial pathogens contribute to food safety issues, including *Escherichia coli* (e.g., O157:H7), *Listeria* spp., *Campylobacter* spp., *Shigella* spp., *Clostridium* spp., *Staphylococcus aureus*, *Bacillus cereus*, and *Vibrio* spp. [31,32]. However, *Salmonella* spp. and *Campylobacter* spp., which are commonly associated with poultry and poultry products, are the leading cause of foodborne illness, with *Salmonella* infections reported to be a leading cause of mortality [31]. *Salmonella* is a Gram-negative non-spore-forming bacillus belonging to the *Enterobacteriaceae* family, with a diameter of 0.75–1.5 µm and a length of 2–5 µm [33]. *Salmonella* spp. are mostly chemotrophic and often possess peritrichous flagella, with the exception of *S.* Gallinarum and *S.* Pullorum, non-motile bacteria highly pathogenic in poultry [33,34]. *Salmonella* spp. are considered non-fastidious microorganisms, capable of colonizing and multiplying under a wide range of environmental conditions. They are classified into two main species: *Salmonella enterica* and *Salmonella bongori*. *S. enterica* comprises over 2500 serovars, with about 80 commonly associated with salmonellosis in animals and humans, while *S. bongori* is further divided into six subspecies, with over 20 serotypes typically linked to infections in cold-blooded animals and, occasionally, humans [33,35]. *Salmonella* spp. are facultative anaerobic bacteria of major concern in agriculture (especially poultry production) and the food industry [33,36].

*Salmonella* infection is a zoonotic disease of public health significance worldwide [37,38]. Poultry and poultry products serve as primary reservoirs, with approximately 200 serovars, including *S.* Enteritidis and *S.* Typhimurium, commonly implicated in poultry-related outbreaks [39,40]. *Salmonella* is commonly associated with contaminated food from livestock and poultry sources, such as eggs and raw meat [41,42,43]. Transmission may also occur through contact with contaminated animal-based food, a contaminated environment, or via cross-contamination during food handling [44]. As a zoonotic pathogen, it can be transmitted from animals to humans either through direct contact or consumption of contaminated food, causing various pathologies. *Salmonella* is a significant foodborne pathogen, causing infections in both animals and humans, representing a major public health risk globally, particularly in developing countries [34,45].

**Figure 1:**
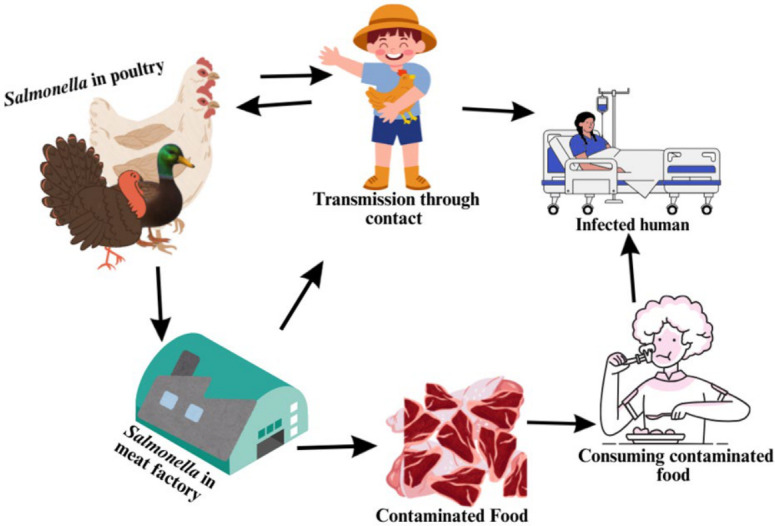
Transmission pathways of *Salmonella* within the poultry value chain.

The U.S. Center for Disease Control and Prevention (CDC) estimated that *Salmonella* causes approximately 1 million infections, 19,000 hospitalizations, and 380 deaths annually in the United States alone [45]. Globally, it is estimated that 93 million *Salmonella* gastroenteritis and 155,000 associated deaths are reported annually [19]. In a 2024 report, the CDC documented a *Salmonella* outbreak in the United States linked to backyard poultry that alone resulted in 470 cases, 125 hospitalizations, and one death. Salmonellosis typically causes diarrhea, fever, abdominal cramps, and vomiting. These symptoms may be more severe, particularly in immunocompromised individuals such as the elderly, infants, and pregnant women [45]. The control of *Salmonella* is further complicated by the recent emergence of multidrug-resistant strains [46]. Transmissions to humans can also occur through cross-contamination at various points from farm to fork (Figure 1). Figure 1 illustrates the transmission of *Salmonella* from poultry to humans through direct contact or with the consumption of contaminated meat.

In summary, *Salmonella* spp. comprises multiple serotypes that cause various infections in both poultry and humans, leading to significant global economic losses, high treatment costs, and mortality. Therefore, effective control strategies are urgently needed to reduce *Salmonella* and ensure a *Salmonella*-free poultry value chain, thereby improving food safety.

## 3. Current Control Methods for *Salmonella* in Poultry

*Salmonella* control remains a top priority for poultry producers, the poultry industry, and public health authorities. When *Salmonella* was discovered in the 19th century by Theobald Smith, early livestock farming had limited knowledge of control strategies, leading to a severe impact of salmonellosis on poultry farms. In fact, between the 1950s and 1970s, blood testing followed by the culling of positive birds was used to eliminate *S.* Gallinarum and *S.* Pullorum from the poultry sector in many countries. Since the bacteria are vertically transmitted, the elimination of positive breeder birds became impossible. This contributed to the emergence of *S. enterica* and *S*. Typhimurium as major foodborne pathogens that spread globally between the 1970s and 1990s [47]. This prompted the need for holistic control from farm to fork, with the introduction of regulatory and industrial interventions such as the UK Zoonoses Action Plan (ZAP) [48] and the EU Zoonoses Regulation (EC) No. 2160/2003 [49], all aimed at controlling *Salmonella* from farm to fork. Despite these efforts, the eradication of *Salmonella* spp. is unrealistic and has remained elusive, thus necessitating the adoption of modern control strategies. These include the use of antibiotics, vaccination, biosecurity measures, management practices, feed supplements, and other interventions in slaughterhouses and meat processing plants.

Biosecurity refers to a wide range of measures, procedures, and policies used to protect animals, humans, and the environment from harmful biological agents, including bacterial infections and contaminations. Although effective biosecurity will reduce bacterial transmission between sources, factors such as animal transport from farms to slaughterhouses, lairage conditions, slaughter practices, and poor biosecurity in slaughterhouses can promote *Salmonella* cross-contamination and infection [50,51]. Key biosecurity measures include restricted farm access, rodent and pest control, housing system clean-out between production cycles, and implementation of the all-in/all-out production system on a farm to prevent *Salmonella* cross-contamination and subsequent transmission [15,52,53]. Additional biosecurity interventions such as farm management practices, hygiene and sanitization, flock monitoring and quarantine, and feed and water treatment contribute significantly to the control of *Salmonella* in poultry farms [54]. Although these measures are effective for short-term prevention, they are sometimes inadequate as long-term control methods. While biosecurity offers the advantage of reducing antibiotic use, the complexity and cost of implementation, particularly for small-scale farmers, often lead to non-compliance or partial adoption [15,16]. The cost of biosecurity in poultry can be considerably high, averaging 3.55 euro cent per bird for broiler producers and 75.7 euro cent for hatching egg producers. These correspond to 0.10 euro cent per bird per rearing day and 0.27 euro cent per bird per rearing day, respectively [55]. Moreover, human behavior and attitudes may influence the poor adoption of biosecurity protocols [56].

Vaccination serves as another prophylactic method for controlling *Salmonella* in the poultry industry. Both live attenuated and inactivated vaccines are commonly used to reduce *Salmonella* spp. colonization and shedding, thereby minimizing the risk of cross-contamination. Live attenuated vaccines induce a stronger and more robust immune response, while inactivated vaccines are considered safer. However, vaccine choice depends on farm conditions and risk assessment [47]. Although vaccines will reduce the over-reliance on antibiotics, some limitations remain. Aside from the cost of using vaccines, some vaccines may offer only short-term protection and thus limited value. Furthermore, only a few high-quality modern vaccines provide protection against multiple *Salmonella* serovars, but this does not completely eradicate *Salmonella*; hence, the protection against one serovar may create an ecological niche for the emergence of other harmful serovars. For instance, the successful 20th-century US eradication campaign targeting *S.* Gallinarum and *S.* Pullorum in chickens has left a void that was much later on filled by *S.* Enteriditis, a previously rare serotype that became the leading cause of human salmonellosis [47]. Additionally, vaccine failures may arise from host-related factors such as interference from maternal-derived antibodies, stress, an immunocompromised flock, or incorrect dosing, administration route, absence of a booster dose, and low vaccine potency due to poor storage conditions [57].

Other farm-level interventions, such as the use of acidifiers, organic acid treatment, heat-treated feed, prebiotics, probiotics, postbiotics, phytobiotics, and synbiotics, also help to control the proliferation of *Salmonella* [33,54,58]. Prebiotics are compounds supporting the growth of probiotics, while postbiotics are bioactive compounds produced by probiotics. Together, these feed ingredients or additives improve gut health and disease resistance. While probiotics often receive the most emphasis, their effectiveness is challenged by strain specificity or variable efficacy, inconsistent outcomes, diminishing efficacy under high bacterial load, and interaction with other feed additives. Additionally, their cost limits their accessibility to small-scale farmers. Some probiotics are also heat- and oxygen-sensitive, thus compromising their field efficacy [59]. Phytobiotics are plant-derived natural compounds used in animal nutrition, particularly in poultry, to improve animal health, digestion, immunity, growth, and performance. Despite their benefits, some may be toxic if improperly dosed by untrained farmers. High variability in active ingredients, the forms of phytobiotics, their compatibility with current diets, and differences in birds’ growth stages pose challenges, especially for smallholders [60,61]. These issues are likely to hinder the broader adoption of phytobiotics by the poultry sector for *Salmonella* control.

**Figure 2:**
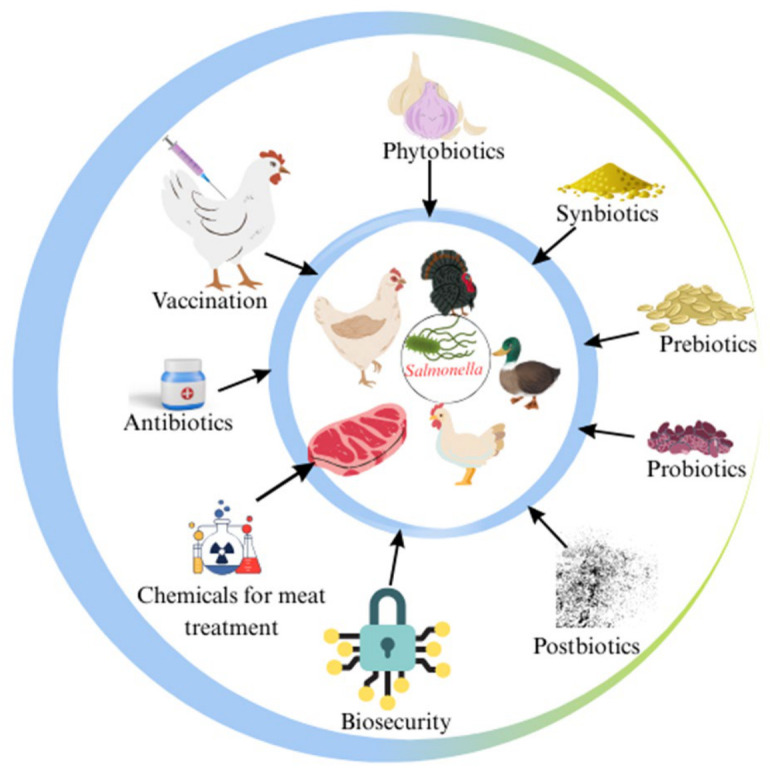
Current methods for the treatment of *Salmonella* contamination or infections in the poultry industry.

Another widely used method for *Salmonella* control in poultry is the application of antibiotic treatment. However, the misuse of antibiotics has led to the emergence of a multitude of antibiotic-resistant *Salmonella* strains [62,63], raising public health concerns [64]. The persistence of antibiotic residues in foods and the recent rise of multidrug-resistant *Salmonella* strains have thus intensified the challenges facing the control of *Salmonella* [17]. Additionally, in slaughterhouses and meat processing plants, chicken carcasses are often treated with chemicals such as peracetic acid, chlorine, post-steam antimicrobial dips or spray, preservatives, and heat treatment to reduce bacterial (e.g., *Salmonella*) contamination [65]. While effective, these methods may reduce the nutritional, sensory, and organoleptic quality of meat, as well as cause the presence of chemical residues in meat, thereby reducing consumers’ acceptability for poultry meat products [12,13,66]. This underscores the urgent need for alternative strategies to control *Salmonella* within the poultry value chain. Figure 2 illustrates various *Salmonella* control strategies along the farm-to-fork continuum.

In summary, although biosecurity reduces the use of chemicals and antibiotics during poultry production, the complexity and cost of implementation, along with human perceptions, often lead to non-compliance and poor adoption. The emergence of resistant or super bacterial variants, higher cost, and vaccine failures are some of the major drawbacks associated with the use of vaccines and antibiotics in poultry production. High *Salmonella* loads and strain specificity can result in inconsistent outcomes or the failure of probiotics. Probiotics may also interact with food additives and environmental agents, reducing their effectiveness. Insufficient knowledge regarding active ingredients, along with the presence of potential toxins in phytobiotics, can disadvantage even skilled farmers. Chemical and antibiotic residues in meat products will affect consumer acceptance. These drawbacks are indeed causes for concern and highlight the need for novel, sustainable biological approaches.

## 4. Phage Types Associated with *Salmonella* Enteritidis or *Salmonella* Typhimurium

*Salmonella* strains can be differentiated by their susceptibility and lysis patterns to specific phages, and this principle underlies phage-typing methods used in epidemiological investigations. For *S*. Typhimurium (e.g., DT9, DT12, DT21, DT104, DT193), definitive phage types are determined using the Anderson typing scheme, which employs a diverse panel of phages [67,68] (Table 1). In contrast, *S.* Enteritidis (e.g., PT1, PT4, PT6, PT8, PT13) is defined by characteristic susceptibility patterns to a panel of Ward-typing phages [69] (Table 1). This implies that the same general principle applies to both serovars, but each has its own standardized phage-typing system for identifying and comparing strains in public health investigations.

## 5. Application of Bacteriophage in *Salmonella* Control

Bacteriophages are viruses that infect and kill bacteria by using the bacterial DNA replication machinery to replicate themselves, killing the bacteria in the process. Phage infection is mediated by the presence of specific receptors that enable recognition and attachment to the bacterial host cell surface. For instance, the expression of AmpC β-lactamase, a bacterial enzyme responsible for resistance to ampicillin, has been associated with enhanced susceptibility to phage infection via receptors such as OmpA and OmpC [71]. Receptors reported for phage attachment include OmpC (major outer membrane protein), FhuA (ferrochrome outer membrane transporter), lipopolysaccharide O antigen, and surface glycoconjugates [72,73]. According to Álvarez et al. [74], phages recognize host cell surfaces through these receptors, followed by adsorption and penetration, during which phage DNA is injected into the bacterial cell. This is followed by replication, synthesis of viral parts, and assembling of new phage particles, culminating in host cell lysis and release of progeny phages, ultimately killing the bacterium. Based on the number and diversity of tail fibers and receptor-binding structures, phages can be classified as broad-spectrum (broad host range), capable of infecting multiple bacterial species, or narrow-spectrum (narrow host range), which infect only a limited number of bacterial species. Consequently, their potential as antimicrobial agents has been increasingly explored for the control of bacterial infections and contaminations. Phage therapy is considered a novel biological method that has recently gained significant interest for bacterial control. Evidence of its potential application has been reported in various fields, including the poultry sector, to control bacterial contamination. Although several studies have explored the effectiveness of phages against bacteria of importance in poultry, there remains limited knowledge on their application, specifically against *Salmonella*, within the poultry value chain. This underscores the need to review existing data and assess the potential and limitations of phage use against *Salmonella* in the poultry industry.

### 5.1. Pre-Harvest Applications (On Farms)

*Salmonella* is a major foodborne pathogen that poses a significant threat to poultry production at the farm level. Traditional control methods applied in the poultry sector often prove challenging and yield undesirable outcomes. However, phage-based treatment offers a promising biological alternative capable of controlling *Salmonella* infections in poultry farms [23,75,76,77]. The effectiveness of phages has been demonstrated at various stages of the poultry production cycle (from hatchery to harvest) by reducing *Salmonella* to acceptable levels. However, the efficiency of phage-based treatment on poultry at the pre-harvest stage largely depends on several factors, including the phage strain and environmental factors, phage concentration or dose, and the mode of delivery (Table 2).

A study conducted in Egypt on six poultry farms showed that poultry birds (ages: 3–28 days) treated with Sal-1 phage had significantly lower *Salmonella* loads (10^2^ CFU), including antibiotic-resistant strains, compared to the untreated group (10^4^ CFU) [78]. While the phage-treated group exhibited no mortality and reduced *Salmonella* levels, the control group suffered severely from *Salmonella* [78]. In another study from Brazil, poultry litter was contaminated with *Salmonella* (10^5^ CFU/mL) and treated with phage SM1 (10^6^ PFU/mL), which showed a >99.9% reduction in *Salmonella* load [79]. While the bacterial reductions by the phage strains are attributed to the phage lysis of the bacterial cells, killing them in the process, the low mortality could be attributed to the reduction in *Salmonella* in the treated group, as lower infection levels are associated with lower mortality. Similar reductions in *Salmonella* spp. in birds after phage (different phage strains) treatment have been reported in Pakistan [80], Spain [81], the USA [82], China [83], and Thailand [28,84,85] (Table 2). However, outcomes vary across these studies, likely due to differences in phage strains and environmental factors such as temperature and pH.

Different phage strains react differently to environmental factors or the application conditions, influencing phage activity. A study in Taiwan reported that polyvalent phage, vB_SalS_KY05 (*Tequintavirus*) applied against *Salmonella* in poultry, was stable at pH 4–10, at 40 °C, and in water for up to 7 days [86]. Similarly, in the People’s Republic of China, phage S55 was stable at pH 4–11 and a temperature of 40–60 °C for 1 h, and effectively reduced *S*. Pullorum and *S*. Enteritidis on chicken skin at 4 °C and 25 °C [87]. However, in other studies in Kenya and China, *Salmonella* phages isolated remained stable at only pH 5–9 and temperatures of 25 °C to 42 °C and 60 °C, respectively, and were capable of reducing multidrug-resistant *Salmonella* in poultry farms [88,89]. These differences in stability may be attributed to the different phage strains used in these studies, suggesting that some phage strains can be more tolerable or stable than others in real-world applications. For instance, in China, phage STP4-a exhibited stability in stimulated stomach and intestinal environments and effectively controlled *Salmonella* in chickens when applied as a pretreatment, achieving a complete *Salmonella* eradication within 14 days [90]. The overall stability profiles of these phages suggest their suitability for field application. Phages may survive in the gastrointestinal tract or other adverse environmental conditions. However, outcomes in real-world applications can still be unpredictable. For instance, in Ecuador, a phage cocktail reduced preformed *Salmonella* biofilm by 39.9%. However, under commercial field conditions in turkey farms, no *Salmonella* was detected in either of the treated or control groups, making efficacy inconclusive [91]. This highlights that, in a real-world application, phage efficacy may be unpredictable, emphasizing the need for optimization, phage protection (encapsulation), and field validation prior to large-scale use. Phage encapsulation may offer better protection of phages against application conditions. In India, an in vitro chicken gastrointestinal model demonstrated significant *Salmonella* reduction using an encapsulated phage cocktail, which enhanced phage stability at pH 2.5 for 90 min. In contrast, the free phage cocktail was inactivated at the same pH within 15 min [92]. This implies that the survivability of phages in birds can be enhanced through microencapsulation, protecting them from harsh environmental conditions and enabling their controlled release [82,93]. Although phages can penetrate and kill bacteria and disrupt biofilm, variability under field conditions remains a significant challenge. These findings highlight the key factors to consider when applying phages on poultry farms, including environmental conditions such as pH, temperature, and salinity, as well as the concurrent use of chemicals on farms, which may affect scalability, leading to unpredictable outcomes and increased production costs. Therefore, it is worth noting that the reductions observed under experimental conditions are not conclusive evidence that phages are ready for industrial application as antimicrobial agents, as environmental conditions may differ (unpredictable environmental conditions) in real-world settings. For instance, the gastrointestinal tract may present variable conditions, including unfavorable pH and temperature, which can affect phage viability during industrial application. Therefore, further investigation and optimization are required for effective use.

Another factor observed in this study that influences the effectiveness of phage treatment against *Salmonella* in poultry production is the phage concentrations or doses used. In the UK, a phage cocktail delivered in feed to 672 broiler chickens at different dosages over 42 days reduced *Salmonella* colonization and had a positive impact on growth and performance [94]. However, varying results were observed depending on the dosage, with the lower phage concentration completely eradicating *Salmonella*. For instance, while no *Salmonella* was detected at 10^5^ PFU/day, *Salmonella* (4 × 10^2^ CFU/g) was detected at 10^6^ PFU/day and 10^7^ PFU/day [94]. In a similar study in Taiwan, chickens were challenged with *S.* Typhimurium, and phage vB_SalS_KY05 (*Tequintavirus*) was administered via drinking water at either a low dose (10^5^ PFU/mL) or a high dose (10^8^ PFU/mL). The low-dose treatment reduced splenic *Salmonella* and improved the albumin-to-globulin ratio, as well as beneficial bacteria (e.g., *Lactobacillus crispatus* and *Blautia coccoides*). In contrast, the high dose promoted phage-bacteria coexistence and increased harmful bacterial species (e.g., *Erysipelaclostridium*) [86]. This suggests that a lower dose can, in some cases, be more effective. In contrast, another study in the UK evaluated a phage cocktail delivered via drinking water at different concentrations (3 × 10^5^ PFU/day and 3 × 10^6^ PFU/day) for 28 days to reduce *Salmonella* in 240 laying hens aged 56 weeks. The results showed that the higher-dose treatment reduced *Salmonella* to 0.00 log CFU/g in broilers and led to a significant (*p* < 0.01) 60% *Salmonella* reduction in eggs [95]. Similarly, in the Republic of Korea, laying hens challenged with *S*. Gallinarum and treated with phage (10^8^ CFU/g) at different amounts (5 mg/kg feed and 10 mg/kg feed) showed a reduction in *Salmonella* levels in the excreta on days 3, 7, and 14. It was observed that the 10 mg/kg feed treatment on day 7 resulted in a significantly greater reduction compared to the 5 mg/kg feed treatment [96]. These findings suggest that there is no universal dose rule for phage application. Instead, outcomes depend on the interplay of phage-host dynamics, replication rates, resistance, and lysis behavior. In some cases, lower phage doses may better align with optimal infection dynamics than higher doses. Differences in experimental conditions, phage adsorption and replication rates, bacterial growth rate, lysis inhibition, and resistance selection may explain these disparities.

This study also found that the success of phage therapy in poultry farms depends on the application method (the mode of delivery) and the medium through which phages are applied [23,97,98]. For instance, in a study in Pakistan, phage BP13076 significantly reduced *Salmonella* on table and breeder eggs via the immersion method at MOI 1000 (by 2.58 and 2.6 log CFU/egg at 4 °C and 25 °C, respectively) and MOI 100 (by 2.27 and 2.58 log CFU/egg at 4 °C and 25 °C, respectively). In contrast, spraying the eggs with the same phage under the same conditions eradicated *Salmonella* at MOI 1000 and significantly reduced *Salmonella* at MOI 100 within 24 h [75]. This implies that application methods can influence the outcome of phage treatments; however, variations are still observed across treatment temperatures within the same application method, with greater bacterial reductions recorded at lower temperatures. These varying outcomes can pose a challenge for phage validation for industrial applications, as they may require customization of phage products for use at different stages of the poultry production chain, which can be time-consuming and increase production costs. Similarly, in China, pathogen-free chicken eggs were experimentally contaminated with *Salmonella* and subsequently treated with phage SP02 via spraying to evaluate its effects on *Salmonella* load, hatching rate, chick survival, immune organ indices, intestinal flora, and weight. The treatment effectively eradicated *Salmonella* from egg shells and chicks within 12 h and improved hatching rate, survival rate, and weight gain without causing immune damage [99]. In another study in Poland, an in vivo study of a phage cocktail (UPWr_S134), consisting of three phages (UPWr_S1, UPW_S3, and UPWr_S4), demonstrated degradation of biofilms formed by *Salmonella* Enteritidis strain 327 lux and ATCC 13076. It also reduced and, in some cases, completely eradicated *Salmonella* on the surface of poultry drinkers and decreased the overall bacteria load in experimentally infected chickens, indicating the potential of phages for controlling *Salmonella* in poultry farms [100]. A similar study in Turkey recorded reductions in *Salmonella* levels in drinking water, on shavings, and on plastic surfaces by 2.80 log_10_, 2.30 log_10_, and 2.31 log_10_ units, respectively, using a phage cocktail [101]. While Korzeniowski et al. [100] assessed a more limited farm environment, Evran et al. [101] included multiple farm environmental matrices, which showed that drinking water exhibited approximately 0.5 log unit greater reduction than other farm environments. This may be attributed to the enhanced mobility of both bacteria and phages in water, which facilitates phage adsorption and infection. Thus, the medium of application can significantly influence phage efficacy. A phage cocktail was administered to 200 chicks in France via drinking water as a prophylactic treatment for 6 days, followed by *S.* Enteritidis infection for 7 days. The results showed that the phage cocktail significantly reduced *Salmonella* colonization in the caeca by 10^3^ log CFU up to the fourth day of post-infection [102]. However, *Salmonella* levels rebounded after 8 days of phage treatment [102], likely due to the emergence of a resistant bacterial population that survived and regrew. In Spain, a phage cocktail was evaluated in two experimental setups to determine the safest and most effective delivery site against *S.* Typhimurium in hatcheries. The study found that administration via the amniotic liquid was the safest and most effective in vivo inoculation route, reducing *S.* Typhimurium colonization in directly infected chicks and preventing transmission to unexposed birds [103]. During the late embryonic stage, ingestion of amniotic fluid enables phages to reach the gastrointestinal tract directly, which is the primary site for *Salmonella* colonization. However, this method did not produce a long-term effect. Additionally, lytic phage LP31, isolated from poultry feces, reduced *S.* Enteritidis on metal surfaces (0.951 logCFU/mL) and on chicken feces (2.14 log CFU/mL) as well as biofilms formed by *S.* Enteritidis and *S.* Pullorum [104]. This disparity across different application media could be attributed to variations in the chemical composition and physical properties of the media, as this may affect phage particle movement. Overall, these findings suggest that the mode of phage delivery and target site significantly influence the efficacy of phages against *Salmonella* in the poultry value chain. The use of phage cocktails likely enhances effectiveness due to synergistic interactions among different phage strains [105,106]. However, long-term efficacy may depend on the specific composition and operational conditions, while prophylactic application may offer more sustained benefits. Since the application method will be influenced by farm protocol as well as farm conditions, this should be considered carefully for successful implementation.

The synergistic effect of phage cocktails, phages supplemented or combined with antimicrobial agents, and serial treatment were other factors found in this study that may further enhance the efficacy of phages against *Salmonella* in poultry farms. In Thailand, phage WP109 lysed over 90% of *Salmonella* isolates from broilers, while phage WP128 lysed 78.2%. This indicates that disparities in the efficiency of phage-mediated bacterial lysis (lower to higher bacterial lysis) can be observed among different phage strains. However, when used in combination (cocktail), over 99% lysis was observed [77], highlighting the synergistic effect of the two phage strains. In a similar study, 30–90% reductions in multidrug-resistant *Salmonella* along the broiler value chain were reported following the application of phage cocktails [84], which were up to 9% lower than those achieved with the WP109 and WP128 phage cocktail. Another study in Iran demonstrated a significant reduction in *Salmonella* in infected broilers following treatment with a phage cocktail, which also led to an increase in weight gain and low mortality [107]. These differences may be attributed to the use of different phage strains that facilitate synergistic activity. Phage strains have different bacterial receptor recognition ability; hence, their combination leads to enhanced synergistic effects and an increased chance of bacterial recognition and attachment.

Phage efficacy can also be improved by combining them with other compounds such as antimicrobials, probiotics, or organic acids. Choi et al. [108] found that a phage cocktail (SLAM-phiST45 and SLAM-phiST56) suppressed the growth of *S.* Typhimurium in a simulated chicken gastrointestinal tract, although some pathogenic strains survived, evolved, and proliferated. However, when combined with the probiotic *L. reuteri* J2M1, there was a greater reduction in *S.* Typhimurium and a significant increase in beneficial microbes. Although it could be inferred that the probiotic contributed to the significant reduction in *Salmonella*, other factors, such as the physiological state of the bacteria and incubation temperature, may influence the outcome, warranting further investigation. In Korea, a similar study demonstrated the synergistic activity of phages and probiotics, showing greater *Salmonella* reduction in a chick model compared to a phage-only treatment [109]. Although such combinations may expand host range and reduce the likelihood of phage resistance, they do not guarantee long-term efficacy. Applying phages (MOI 10) concurrently with the antibiotic gentamycin (concentration 0.582 µg/mL) significantly (*p* = 0.02) reduced *Salmonella* in broilers [110]. Similarly, in Poland, a phage cocktail (phage vB_SenM-2 and phage vB_Sen-TO17), applied alone or in combination with ciprofloxacin, reduced *S.* Typhimurium within 24 h, with no significant differences between treatments. However, in the treatment with the cocktail alone, the efficacy of the phage cocktail declined after 48 h [111]. Organic compounds or organic acid supplementation can also enhance phage efficacy. In China, phage vB_SalP_NW15, combined with cinnamaldehyde and administered in vivo, reduced bacterial load, alleviated histopathological damage in the heart and liver, and improved survival rates of chickens [112]. In another study, chickens challenged with *Salmonella* Enteritidis and subsequently treated with a feed supplemented with phage (1000 g/t) and organic acid (acidifier A) showed a reduction in *Salmonella* levels, increased beneficial bacterial population, and improved body weight, feed intake, feed conversion ratio, and meat quality [113]. These supplementations will significantly reduce production costs and increase productivity. However, another challenge could be the effect of the chemical dosage on the animal, as this may adversely affect its physiological being and performance.

With the serial treatment, a phage cocktail was applied in simulated poultry farms in Spain following cleaning and disinfection with carvacrol, resulting in a complete elimination of *Salmonella* [114]. This outcome may be attributed to the sequential and synergistic effects of the treatments: cleaning removes dirt and biofilms, exposing bacterial cells; disinfection eliminates exposed cells; and phage treatment targets surviving bacteria. In a related study in Spain, lytic phages (vB_Si_CECAV_FG009, vB_Si_CECAV_FG017, vB_Si_CECAV_FG029, and vB_Si_CECAV_FG030) applied between cleaning and disinfection reduced antibiotic-resistant *Salmonella* prevalence from 100% to 36% and subsequently to 0% after disinfection [115]. Overall, these findings suggest that sequential and combinatory treatments (e.g., cleaning, followed by disinfection and then phage treatment) can be highly effective in controlling *Salmonella*, particularly in critical transmission points such as water systems. A summary of phage applications against *Salmonella* in poultry production is shown in Table 2.

In summary, phages are effective in controlling *Salmonella* spp. at all stages of poultry production (from hatcheries to harvest). This novel biological method shows some promising results and may gain public acceptance that could improve food safety and public health. However, various factors may influence the practical success of phage therapy in poultry farms, including the phage strain, environmental conditions, phage concentration, phage synergy, and mechanisms of infection. Different phage strains have different mechanisms of infection. While higher phage concentrations increase the ratio of phages to bacteria, increasing the likelihood of infection, lower phage concentrations may limit the chance of infection. However, the findings in this study showed that a lower concentration can be more effective. Phage cocktails or combinations with antimicrobials may provide synergistic effects; however, dosage requires optimization for industrial application. External factors such as pH, temperature, and salinity also affect the physiological dynamics of phages. Therefore, validating phage formulations under these conditions is crucial before phage application against *Salmonella* spp. in poultry farms, leading to a reduction in production costs, limiting treatment failures, and increasing productivity.

### 5.2. Post-Harvest Applications (Processing Plants)

In most cases, harvested birds are sent to slaughterhouses and meat packaging plants for processing and transformation into poultry products for the consumer retail market. In poultry slaughterhouses and meat processing facilities, the environment and working conditions create a high risk of bacterial proliferation and cross-contamination, including from *Salmonella* spp. Methods such as physical and chemical (e.g., chlorine) treatments are often used to reduce bacterial contamination. However, these methods may affect the physicochemical and organoleptic properties of meat and reduce consumer acceptability.

As a result, the application of phages can be considered as a biological alternative that reduces bacterial contamination while maintaining product quality and consumer acceptability. Studies have shown that *Salmonella*-specific phages are effective in controlling *Salmonella* contamination in poultry meat [116,117,118,119]. Findings from China [120], Canada [121], Germany [104], the Republic of Korea [122], Thailand [23], and Egypt [123,124] have demonstrated notable *Salmonella* reductions in meat after phage application (Table 2). However, differences were observed among the results reported by these studies, which is likely attributed to the variations in phage strains, phage concentrations, treatment duration, medium conditions (e.g., temperature, salinity, and pH), and the type of food matrix considered. These factors are common in the meat processing industry and can influence the efficacy of phages against *Salmonella* spp. in poultry meat during harvesting or processing.

Phages have demonstrated some excellent efficacy in reducing or even eliminating the bacterial load on eggshells. However, the efficiency of phages against *Salmonella* on eggs is influenced by the phage strain, concentration, application method, and the duration of treatment. For instance, a study in India applied *Salmonella* phage PSE5 at 0.01 MOI using an immersion method on 40 eggs sourced from local markets. This led to a reduction in antibiotic-resistant *Salmonella* strains by 2.0 × 10^6^ CFU/mL within 2 h after treatment [125]. Although a significant reduction was observed, this study is limited by the low phage concentration (without concentration diversification) used. In another study in China, phages sp11241 and 8–19 applied (by the spraying method) at MOIs of 1, 100, and 1000 at 4 °C, completely eliminated *S. enterica* on chicken eggs after 3 h and 6 h at MOI 100 and after 2 h and 5 h at MOI 1000, respectively [126]. The disparity in the outcomes could be attributed to differences in delivery method and phage concentrations used in the two studies. However, in raw chicken meat, maximum reductions of 3.17 and 2.69 log_10_ CFU/mL were observed after 12 h with phage sp11241 and 8–19, respectively [126]. In another study at 4 °C and 25 °C, phage LPST94 reduced *Salmonella* in chicken breast by 3 log_10_ CFU/mL within 48 h at MOIs of 1000 and 10,000 [127]. These imply that differences in phage strains and composition between meat and eggs can lead to different outcomes. Although similar reductions are observed between the study by Islam et al. [127] and Sun et al. [126], the phage strain used by Sun et al. appeared to be more efficient, achieving comparable reductions in *Salmonella* at a lower MOI. Phage Salmp-p7, applied at an MOI of 0.001, effectively reduced *S.* Enteritidis in liquid eggs, inhibited biofilm formation after 7 h, and remained active under extreme conditions, including pH 4–12 and temperatures between 30 and 60 °C [128]. However, variations in bacterial physiological state over time may influence phage efficacy. For instance, in the USA, the application of *Salmonella* phage on chicken breast at 4 °C resulted in significantly different reductions in *Salmonella* Typhimurium DT104 counts at 0, 1, 24, and 48 h [129]. Also, these findings suggest that certain phages can remain viable under harsh processing conditions. During phage application, stability is a factor for producers who aim to maximize production. Therefore, phages with high stability potentials are mostly preferred for validation for commercial use.

This study also found that the food type (type of poultry meat) can influence phage efficacy. The food matrix plays a crucial role in phage effectiveness. Variations in the nutritional composition of different food matrices may influence the rate of bacterial growth. Food matrices favoring bacterial growth and promoting rapid bacterial growth and proliferation may reduce the phage-to-bacteria ratio and thereby reduce phage efficiency. Additionally, the type of food matrix may also influence the movement of phage particles, temperature, salinity, and pH, as these can degrade the phage capsid, rendering them inactive. For example, compacted food may restrict the movement of phage particles, limiting their rate of penetration into bacteria. According to Islam et al. [127], phage efficacy varies depending on the food matrix and environmental conditions. Moon et al. reported that phage treatment in tryptic soy broth reduced *S*. Typhimurium JWC-3001 by 5 log CFU/mL at a lower MOI (≥1.7), whereas a higher MOI was required to achieve similar reductions in chicken meat [130].

Furthermore, this study revealed that phages can also be used in cocktails or combined with other antimicrobials to control *Salmonella* during the post-harvest stage of poultry production. A phage cocktail (phage SPHG1 + SPHG3) applied for 48 h at MOIs of 100 or 1000 significantly reduced *S*. Typhimurium by 4.2 log_10_/sample on chicken breast [123]. However, in the USA, a study showed that combining phages with thymol and carvacrol (1.6% *w*/*v*) significantly reduced *Salmonella* in broiler carcasses [130]. In a similar study in Turkey, a phage cocktail (phage AUFM_Sc1 + phage AUFM_Sc3) applied in combination with nisin reduced *Salmonella* on chicken breast within 48 h. Although a rebound in *Salmonella* was observed, the *Salmonella* levels remained within the acceptable limits [131]. The combined effect of phages and organic acid against *S*. Enteritidis in chicken meat was reported in Thailand [132]. These suggest that combining phages with other antimicrobials could be effective through synergistic activity; however, the chemical compositions of these antimicrobials must be taken into account, as phage activities can vary with media composition.

Although phage cocktails are proven to be effective due to their synergistic effects against bacterial pathogens, phage cocktail training could improve their efficacy by widening their host ranges. A study in Germany evaluated a trained three-phage cocktail (30 cycles according to the Appelmans protocol) for the reduction in *Salmonella* on chicken fillets at 4 °C for 3 days. The trained phage cocktail exhibited an expanded host range (62.5%) compared to the untrained cocktail (37.5%) [133]. Moreover, the trained phage cocktail (at MOI 10 and 100) demonstrated a significantly greater inhibitory effect (100%) within 3–6 h compared to the untrained phage cocktail (25%) [133]. This enhanced performance may be attributed to adaptive infection mechanisms acquired during phage training. This can improve phage recognition of bacterial receptors, facilitating more efficient infection.

Overall, the findings indicate that phages are effective for food safety, although phage training can further enhance their efficacy. However, outcomes vary depending on dose–response relationships, environmental conditions, delivery method, and phage-host interactions. A systematic review from Iran reported that the most commonly used phage dosage for *Salmonella* reduction ranged from 6 to 9 log PFU/mL, with typical application temperatures of 4 °C and 25 °C and treatment durations of 1 to 24 h. Under these conditions, reductions in *Salmonella* up to 10 log CFU/mL have been reported [134]. Temperature and time are critical factors influencing phage activity. Phage efficacy is often greater during the early stage of bacterial inoculation. Lower temperatures may impede the penetration of phage genetic material into the bacterial cells, thereby reducing replication, whereas higher temperatures may prolong the latent period. Notably, a 10-unit increase in temperature has been associated with approximately a 15% increase in *Salmonella* reduction [135]. Similarly, increasing phage concentration by a 5-log unit can lead to near-complete bacterial elimination, while a 2-log unit increase in bacterial load may reduce treatment efficacy by reducing *Salmonella* by only 15% [134]. Both bacterial strain and phage characteristics significantly influence the overall efficacy. A summary of approved phage products and their corresponding countries is shown in Table 3.

**Table 2:** Summary of phage applications in the poultry industry.

Poultry Value Chain	Phage Application Condition	Phage Applications	Country	Reference
Poultry farms	*Salmonella* phage at 10^2–11^ phage-forming units (PFUs) by oral administration	Reduced *Salmonella* load in SPF broiler chicks at day 3, greater reduction at day 5, and complete elimination at day 7.	Egypt	[135]
	Salmo FREE^®^ (10^8^ PFU/mL) delivered in water 3 times in production cycle	Reduced *Salmonella* and mortality in broiler farms with no effect on production or production parameters.	Colombia	[136]
	*Salmonella* phage at 10^8^ PFU/mL and 10^3^ PFU/mL	Reduced *S.* Infantis and *S*. Enteritidis in poultry farm environment by 4.55 log CFU/mL and 3.85 log CFU/mL, respectively, after 5 days.	Spain	[81]
	Phage ϕSET1 (Bs), ϕSET2 (Bs), ϕSET3 (Bs)	Effective against multidrug-resistant *Salmonella* from poultry farms.	Egypt	[137]
	Phage WP109 (Bs), 110 (Bs), 128 (Bs) and their cocktail (5 log PFU, MOI 10^2^)	Significantly reduced *S*. Enteritidis and *S*. Typhimurium from broiler farm environment (farm bedding materials including husks) within 6 h.	Thailand	[84]
	Phage KP001(Bs), KP005(Bs), WP110(Bs) (9 log PFU/mL, MOI 10^3^)	Completely eliminated *Salmonella* from broilers within 20 days in a pilot study.	Thailand	[28]
	Phage CKT1 (Bs for only *Salmonella*) at 10^8^ PFU/chicken	Controlled vertical transfer by significantly reducing *S.* Pullorum in reproductive tract, eggs, and breeding environment.	China	[83]
	A phage cocktail (3 doses, 10^7^ PFU/mL).	Completely eradicated *Salmonella* in broiler farm environment after 16 days.	Thailand	[85]
	*Salmonella* phage cocktail	Reduced *S*. Enteritis and *S*. Typhimurium in broilers at days 7, 14, and 21.	Iran	[138]
	Phage S4lw (Bs) and D5lw (Bs) and cocktail (MOI 100 or MOI 1000)	These phages reduced *S*. Enteritis and *S*. Typhimurium in poultry by 1.7–3.4 log at 2–6 h and their cocktail completely inhibited *S*. Enteritis and *S*. Typhimurium at 14 h at 25 °C.	USA	[82]
	Phage cocktail at 10^10^ PFU/mL	Decrease *Salmonella* Enteritidis in roasters and their environment to 11–26%.	Argentina	[139]
	Phage SEpBS-1 (Bs for only *Salmonella*) at 10^11^ PFU/mL	Decreased *Salmonella* in broiler chickens by 40% at day 14.	Thailand	[140]
	*Salmonella* phage and phage-synbiotic combined treatment	Significantly reduced *Salmonella*, proved antioxidants, immunity, feed conversion ratio, growth, and weight gain.	Egypt	[141]
	Feed supplemented with phage at 5 pp and 10 pp.	Significantly reduced *S*. Typhimurium in the spleen, oviduct, caecum, and excreta in laying hens within 7 days after *S*. Typhimurium challenge.	Korea	[142]
	Phage vB_SalP_LDW16 at 60 °C and pH 5–9	Reduced *Salmonella* in a chicken model.	China	[88]
	Phage SM1 (10^6^ PFU/mL)	A >99.9% reduction in *Salmonella* in poultry litter.	Brazil	[79]
	A phage cocktail delivered via feed to 672 broiler chickens at over 42 days	No *Salmonella* was detected at 10^5^ PFU/day, *Salmonella* (4 × 10^2^ CFU/g) was detected at 10^6^ PFU/day and 10^7^ PFU/day.	UK	[94]
	A phage cocktail delivered via drinking water at 3 × 10^5^ PFU/day and 3 × 10^6^ PFU/day for 28 days against *Salmonella* in 240 laying hens aged 56 weeks	The higher-dosed treatment reduced *Salmonella* to 0.00 log CFU/g in broilers and led to a significant (*p* < 0.01) 60% *Salmonella* reduction in eggs.	UK	[95]
	Laying hens treated with phage at 5 g and 10 g	A reduction in *Salmonella* levels on days 3, 7, and 14, with 10 g on day 7 recording the highest reduction.	Republic of Korea	[96]
	A phage cocktail (UPWr_S134), consisting of three phages (UPWr_S1, UPW_S3, and UPWr_S4)	Degradation of biofilms formed by *Salmonella* Enteritidis strain 327 lux and ATCC 13076. It also reduced and, in some cases, completely eradicated *Salmonella* on the surface of poultry drinkers and decreased the overall bacteria load in chickens in vivo.	Poland	[100]
	A phage cocktail against S*almonella* in drinking water, on shavings, and on plastic surfaces	Reductions in *Salmonella* levels in drinking water, on shavings, and on plastic surfaces by 2.80 log_10_, 2.30 log_10_, and 2.31 log_10_ units, respectively.	Turkey	[101]
Poultry-based foods (meat)	Pu20 at 10^8–9^ PFU/mL and MOI 1000 by direct inoculation (Bs)	Reduced drug-resistant *Salmonella* in liquid eggs by 1.06 log10 CFU/mL and 1.12 log10 CFU/mL at 4 °C and 25 °C, respectively in 24 h.	China	[143]
	Five *Salmonella* phages, SEG5 (Bs), STG2 (Bs), STG5 (Ns), STS9 (Ns) SES8 (Ns), at 3 × 10^8^ PFU at 25 °C by suspension application	Reduced *S*. Enteritidis and *S*. Typhimurium on chicken breast by 3.06 log CFU/piece and 2.21 log CFU/piece, respectively.	Japan	[144]
	SPHG1 (Ns) and SPHG3 (Bs) at 8.3 log10 PFU by spotting and MOIs 1000 or 100	Significantly reduced *S*. Typhimurium on ready-to-eat chicken breasts.	Egypt	[123]
	vB_StyS_LmqsSP1 phage at 10^8^ PFU/cm^2^ and at 4 °C in 3 h and for 7 days by direct inoculation	Reduced *Salmonella* on chicken skin by 2 log units.	Germany	[145]
	SE-P3, P16, P37, and P47 at 10^9^ PFU, 4 °C and 25 °C	A complete reduction in *Salmonella* on chicken and chicken skin.	Turkey	[146]
	Five phages (Bs) at 10^9^ PFU and 10 °C for 48 h	Reduced *Salmonella* on chicken meat by 1.4 log units.	Chile	[147]
	PS3-1 phage (Bs)	A significant (*p* < 0.05) reduction in *S*. Typhimurium on artificially contaminated ready-to-eat steamed-chicken breast after 24 h at 7 °C.	Japan	[120]
	Phage cocktail (STP-1, STP-2, STP-3, and STP-4) (Bs for only *Salmonella* strains) at MOI of 10^3^	Reduced *S*. Typhimurium in artificially contaminated chicken breast by 0.9 and 1.2 log CFU/g after day 1 and day 7, respectively.	Republic of Korea	[122]
	Phage cocktail at 10^7^ PFU	Significantly reduced *Salmonella* by 3 log10 and 2.4 log10 units on fresh chicken skin and stainless steel.	Germany	[98]
	*Salmonella* phages SF1, SE18, SS4 at MOI 10^2^ and 10^3^	Reduced *Salmonella* by 1–7 log CFU/mL in chicken egg white and yolk after 30 days.	Canada	[121]
	Phage L223 (narrow host range) applied on chicken breast at 25 °C	Significantly reduced *S*. Typhimurium on artificially spiked chicken breast by 1.24, 2.17, and 1.55 log CFU/piece after 2, 4, and 6 h, respectively.	Bangladesh	[148]
	Cocktail of phage WPX5 (Bs), WPX8 (Bs), and WPX9 (Bs) at MOI 10^4−5^ for 6 h	Reduced *Salmonella* in chicken meat and food contact surfaces (stainless steel, ceramic, polyvinyl chloride (PVC)).	Thailand	[23]
	*Salmonella* phage 2–3 at MOI 10^4^	Reduced *S*. Enteritidis on chicken meat by 92% after 12 h.	China	[120]
	Phage STGO-35 at 4 °C	Achieved a 2.5 log reduction in *S*. Enteritidis on chicken meat.	Chile	[149]
	*Salmonella* phage DT104 at 4 °C	Resulted in significant different reductions in *Salmonella* counts on chicken breast at 0, 1, 24, and 48 h.	USA	[129]

Bs: broad spectrum; Ns: narrow spectrum.

**Table 3:** Summary of approved phage products and their corresponding countries.

Phage Product	Phage Type/Target	Primary Use	Countries Approved	References
SalmoFresh	Lytic phages for various *Salmonella* serovars (e.g., *S*. Typhimurium, *S*. Enteritidis)	Food safety (poultry/meat)	USA (FDA GRAS)	[150]
Phage guard S^™^ Phage guard S5c	Phage or phage cocktail against *Salmonella* spp.	Poultry/food processing	USA (FDA GRAS, U.S. FDA), Canada, Australia (FSANZ), India, Brazil, Netherland, Switzerland, Chile, Egypt (NFSA), Dubai, Vietnam, Costa Rica	[151,152]
INSPEKTOR^®^ Phage in	*Salmonella* spp.	Poultry treatment	Brazil	[152]
BAFASAL™	*S*. Enteritidis	Poultry feed additive	All EU member states, Brazil, India	[153]
Intestifag	*S. enterica*	Treatment of enteric infection	Ukraine	[152]
GastroPHAG	*S*. Typhimurium, *S*. Newport, *S*. Agona, *S*. Enteritidis, *S*. Java, *S*. Moscow, *S*. Paratyphi B	*Salmonella* infections	Uzbekistan	[152]
Fhagesti, Septaphage, Travelphage™, ENKO bacteriophage	*Salmonella* spp.	*Salmonella* infections	Georgia	[152]
Intesti bacteriophage	*S.* Paratyphi A, *S.* Cholerasuis, *S.* Paratyphi B, *S.* Oranienburg, *S.* Enteritidis	*Salmonella* infections	Georgia, Rusia	[152]

FDA: Food and Drug Administration of the United States, GRAS: Generally Recognized as Safe, U.S. FDA: United States Food and Drug Administration, FSANZ: Food Standards Australia New Zealand, NFSA: National Food Security Act.

In summary, meat is a suitable medium for bacterial growth, and properties such as pH, temperature, salinity, or nutritional compositions of meat from different types of birds and body parts influence the efficacy of phages. Phage concentration, strain, mode of delivery, and their combination with other antimicrobials also affect their effectiveness. These factors may hinder the smooth commercialization or adoption of the technology, as phage-based products may require customization to fit particular processing conditions or products, which can increase production cost.

### 5.3. Consumer Goods Applications (Packaging and Preservation)

Poultry meat is rich in valuable nutrients and provides a balanced nutrient profile to meet human nutritional needs. At the same time, it serves as a nutrient-rich substrate for various microbes, making it susceptible to microbial contamination, including *Salmonella* spp. While *Salmonella* contamination can be controlled through various food preservation methods, such as chemical preservatives and other antimicrobial food additives, these approaches often pose health concerns and reduce consumer acceptability of meat products. This has increased the demand for safer food preservation methods and innovative packaging techniques to control foodborne pathogens.

Bacteriophages have emerged as promising alternatives in food packaging due to their specificity, effectiveness, and natural availability [154]. Phage application represents a biological approach that may enhance consumer confidence towards poultry products. When combined with other antimicrobial agents, phages can synergistically control *Salmonella* contamination in chicken meat during storage. For instance, a bacteriological analysis showed that the *Salmonella* count on chicken filets was reduced by 3 log CFU/mL during storage under alginate treatment and 3.59 log CFU/mL under phage treatment on day 6. However, the combined treatment (alginate + phage) achieved a significant reduction of 6.57 log CFU/mL, compared to the control (4 log CFU/mL) [155]. This corresponds to a *Salmonella* reduction of over 69% without affecting the physicochemical properties of the chicken fillets. This could help eliminate *Salmonella* in storage more efficiently while reducing the cost of the intervention. Temperature also plays a critical role in the effectiveness of phage treatment during storage. For instance, *S*. Typhimurium was significantly inhibited (0.3–4.72 log reduction) by PS3-1 phage (MOI 100) at 7 °C, 25 °C, and 35 °C over 24 h in ready-to-eat chicken breast with no bacterial regrowth [120]. During storage, temperature is a critical factor affecting the shelf life of food products. However, some recalcitrant bacteria can still grow and proliferate under extreme temperatures, leading to food contamination. The survival of phages under these conditions during storage is advantageous, as it can promote bacterial reduction, thereby decreasing contamination and subsequent transmission to humans. However, the outcome can be influenced by the phage concentration used, phage formulation (e.g., cocktail), duration of treatment, and the type of food matrix [130,156,157]. According to Wagh et al. [154], recent studies have explored the incorporation of phages by the food industry during the packaging process. Phage-based packaging films and coatings, such as those using phages such as ΦIBB-PF7A, Felix O1 A511, LITEX^TM^P100, and A511, have been applied via sodium alginate, cellulose membranes, poly (lactic acid), and whey protein or pullulan bilayer. These films were reported to have effectively reduced *Listeria* and *Salmonella* in chicken meat and sliced turkey meat [154]. Although some concerns have been raised about the possible migration of phages from packaging materials to food surfaces, no adverse effects on humans have been reported.

In summary, consumers’ acceptability towards foods is influenced by the type of preservatives used in foods or food packaging. While some additives are beneficial, others may leave chemical residues in the final products, reducing their acceptability by consumers. Phages offer a potential solution to this challenge. However, their effectiveness against *Salmonella* in meat packaging depends on factors such as phage strain, concentration, application method, food matrix, physicochemical conditions, and storage temperature. Although these factors are critical and demand studies to evaluate and validate for adoption, this study revealed that there are limited research papers available regarding phage application against *Salmonella* in consumer goods applications (e.g., packaging).

## 6. Regulatory and Safety Considerations

### 6.1. Regulatory Status of Bacteriophage Use in Foods

Although phages have shown promise in reducing the presence of *Salmonella* spp. and their antibiotic-resistant strains along the poultry value chain, some significant challenges still hinder their full implementation. According to Khan and Rahman [62], the main obstacles include an inadequate regulatory framework and limited safety data. Across the United States, the European Union, and much of Asia, phage-based products are regulated under existing biological and medicinal products frameworks (Table 4); however, regulatory pathways, safety criteria, and commercialization routes differ significantly between regions, and industrial practices follow these regulations [158]. Regulatory hurdles are especially prominent for food packaging applications, which may restrict the use of phages in poultry [159]. For instance, with the EFSA’s cautious stance towards providing phage-based products for food safety, the development of such innovations in the European Union has been significantly delayed. In comparison, countries such as Japan and the UK have adopted more progressive regulatory approaches, underscoring the EU’s slower pace in this area [160]. In the United States, any phage-based product intended for use as a food additive must first receive approval through the Food Additive Petition (FAP) process and from the United States Food and Drug Administration [160,161]. However, in spite of the well-established approval pathway, only a few products, such as ListShield^TM^, have successfully completed this approval process. A summary of phage regulatory and safety criteria in different countries is shown in Table 4.

In summary, regulatory hurdles continue to hinder phage development and applications within the food industry. While some countries have seen the value of this innovative solution to boost food safety and fast-tracked their approval, a lengthy bureaucratic process remains the main hurdle towards their broader use by the food sector.

### 6.2. Safety Assessments for Human and Animal Consumption

Though the application of phages on foods has proven safe for human consumption, their full implementation still faces some challenges due to public perceptions about safety. In view of this, proper phage validation before application is required. Studies have confirmed that phages used for therapeutic purposes are generally safe, lacking virulence, lysogeny, or antimicrobial resistance (AMR) genes [162,163]. An in vitro study has demonstrated that phages are safe and have minimal to no side effects on human living cells [23]. Another safety concern is that phage-treated products may contain endotoxins, leftover molecules generated during the production; however, this can be reduced through further purification methods, such as column chromatography. A study evaluating the toxicity and tolerability of a phage-based supplement (15 milligram capsule containing 4 phage strains, namely, LH01, LL5, T4D, AND LL12) found a significant improvement of gastrointestinal disorders over 28 days, with no adverse effects reported among participants [164]. As part of the regulatory process, phages and their production methods must be granted the “Generally Recognized as Safe” (GRAS) status by food safety regulatory authorities [165].

### 6.3. Public Perceptions and Acceptance

A broader use of phages faces numerous challenges, including addressing current public perceptions. For instance, a study in Poland reported an awareness level of 8.9% among household workers, 37.7% among individuals with higher education, and 39.7% among residents of large cities, with an overall acceptability rate exceeding 80% [166]. This may be attributed to a higher level of education and greater access to information among people in large cities. Conversely, a study conducted in Pakistan reported that the perception and acceptance of phages as alternatives to antibiotics in poultry production were over 70%, indicating some real interest in using phages to control *Salmonella*. However, this consumer acceptance depends on factors such as the stage of production considered and public awareness [167]. These observations suggest that public education on phage application would help increase consumer confidence and acceptance. Educational level and place of residence or location are additional factors that may influence phage acceptability.

**Table 4:** Summary of phage applications regulation in different countries.

Country	Regulator	Classification	Safety Criteria	Trial/Access Path	Commercial Hurdle	Reference
United States	FDA (CBER)	Biological medical product	Genomic safety, GMP, endotoxin-free status, preclinical and clinical efficacy	IND (30-day review), allows expanded access	Phase 1–3 trials required	[158,168,169]
European Union	EMA (centralized)	Biological medical product	Genomic safety, quality, lytic phenotype, GMP, endotoxin-free status, environmental risk.	Clinical Trial Authorization, compassionate use	Marketing authorization post-trials, Phase 1–3 trials required	[158]
Asia (China, India, Japan)	NMPA (China, CDSCO (India), PMDA (Japan)	Innovative biological/pharmaceutical product or ad hoc compassionate use	GMP, ethics, basic characterization and risk-based	IND/IIT (China), cPT (India), risk-based (Japan)	Compassionate dominant; trials emerging, no full approvals	[158,168]

CBER: Center for Biologics Evolution and Research, GMP: good manufacturing practice, IND: investigational new drug, EMA: Europe Medicine Agency, NMPA: National Medicinal Product Administration, CDSCO: Central Drugs Standard Control Organization, PMDA: Pharmaceuticals and Medical Devices Agency, IIT: Investigator-initiated trials, cPT: compassionate phage therapy.

## 7. Challenges and Limitations

Phages have recently emerged as promising antimicrobial agents for controlling bacterial contamination in the food industry, particularly during packaging, thereby enhancing food safety and extending shelf life [154,170]. However, several challenges may arise [171]. These can hinder phage application in both clinical and industrial settings, with implications for animal and human health.

The major limitation in the clinical setting is the narrow host range and strain specificity exhibited by some phages. This poses a significant challenge, as it restricts their ability to infect a broad range of bacterial strains [85,165]. Most lytic phages infect only a limited number of closely related strains within a bacterial species, making it difficult to target all clinically relevant isolates with a single therapeutic cocktail [172,173]. Furthermore, the inability of phages to eliminate all co-infecting bacterial pathogens in polymicrobial infections further complicates treatment [172,173]. This limitation can be mitigated by formulating phage preparations as cocktails, which may provide synergistic effects and enhance efficacy against drug-resistant bacteria. Genetic modification of phages is another potential solution that can increase their lytic activity as well as broaden their host range [154,174]. The use of phage cocktails and targeting phage-resistant bacteria will offer viable strategies to overcome this limitation. Overdose may trigger an immune response, including neutralizing antibodies and cytokine release, which may reduce phage efficacy [173]. Additionally, challenges related to pharmacokinetics, formulation, and delivery represent important limitations in clinical applications. These factors may lead to rapid clearance of phages by the reticuloendothelial system, protease-mediated degradation, and limited tissue penetration, particularly in biofilms and deep-seated infections [173].

The effectiveness of phages under industrial or real-world food packaging scenarios remains a concern, as phages may interact with some components of the food matrix, organic matter, and other food additives, potentially limiting their antimicrobial activity. The use of phages in the food processing industry could be challenging, as their activity depends on the concentration of the target bacteria [174]. Since bacterial levels in food processing environments are usually low, this may limit the effectiveness of phages. Phages can be used as biocontrol agents in industrial settings to sanitize food surfaces, equipment, and water; however, their efficacy may vary, and they may not achieve complete eradication compared to heat or chemical disinfectants [175]. This variability may be further influenced by factors such as surface topology, bacterial load, the type of food matrix (e.g., fat and protein content), food additives, and competing microbiota [176]. Additionally, phage-resistant bacteria may develop in the production environment due to repeated or prolonged exposure, thereby hindering effective bacterial control [176]. Continuous exposure can select for phage-resistant bacterial strains through mechanisms such as CRISPR-Cas systems (type I (Cas3), II (Cas9), and III (Cas10)), abortive infection, adsorption inhibition (receptor mutation), injection blocking, and restriction-modification systems, ultimately reducing long-term phage efficacy [175,177]. Furthermore, phage-induced lysis in food systems may affect product quality, potentially reducing texture, flavor, or yield due to the release of intracellular enzymes such as protease and peptidase. Environmental factors such as changes in temperature, pH, humidity, salinity, and exposure to UV radiation can degrade phages, reducing their efficacy against bacteria [23,154]. These various adverse conditions are often more experienced during the processing of meat products. This can reduce the efficacy of phage in the treatment of *Salmonella* spp. outbreaks in poultry, processing, and hospital environments, thereby posing a risk to public health. These limitations can be mitigated through protective coating or microencapsulation techniques using polymers like chitosan and alginate, as well as lyophilization (freeze-drying), shielding phages from adverse environmental conditions and allowing their controlled release to maintain their viability [93]. However, the effectiveness of such methods will depend on the type of materials used [165,178].

Considering the limitations discussed in both clinical and industrial settings, a critical concern is that some phages may carry resistance or bacterial virulence genes, which could be transferred to different bacterial species through transduction [179,180]. This may have broader implications within the One Health framework, as bacteria can transfer virulence or resistance genes to other bacterial populations in the environment, animals, and humans. Such gene transfer can negatively impact animal and public health, as these bacteria can evade treatment strategies and may spread across different sources through cross-contamination. However, this risk can be mitigated by performing whole-genome sequencing prior to application, which facilitates the selection of safe and suitable phages.

## 8. Conclusions

Numerous *Salmonella* serotypes from poultry sources cause infections in both poultry birds and humans. However, *Salmonella* control remains a challenge due to the worldwide emergence of antimicrobial resistance. Although current treatments can be effective in reducing *Salmonella* at the various stages along the poultry production chain, as revealed in this study, they often leave traces of chemicals in final products, reducing consumers’ acceptability. In contrast, phages have emerged as potentially reliable biological alternatives, as they kill bacteria with high specificity. This study showed that phages can be applied at multiple stages along the poultry value chain. However, challenges such as phage-resistant bacteria, environmental conditions, and interaction between phages and food additives may hinder phage efficacy, as varying outcomes were observed in this study. These factors may hamper the full implementation or rollout of phage-based products as potential alternatives to antibiotics for *Salmonella* control in the poultry value chain. In addition, this study demonstrates that phages can be effective in reducing *Salmonella* in poultry and poultry products; however, limited studies have assessed the sensory evaluation of phage-treated products and the use of phages for poultry product packaging. These areas require further investigations. This research also reveals that regulatory hurdles and public perceptions further impede the full implementation of phage-based treatments. While there is some level of public awareness and acceptance, this can be further strengthened through continuous sensitization efforts and education on the importance of phages within the poultry value chain. This study provides a comprehensive contribution to the existing body of evidence that could inform policy decisions on the full implementation of phages as a potential alternative for controlling *Salmonella* spp. in the poultry value chain, with benefits for animal and public health.

## Figures and Tables

**Table 1:** A summary of phage types and the characteristics.

Phage Type	Bacteria	Infecting Phages	Characteristics	References
DT9, DT12, DT21, DT44, DT49, DT66, DT95, DT99, DT104, DT110, DT120, DT135, DT193, DT204c, DT208, U302; well known among >300 definitive phage types and often associated with multidrug-resistance	*S.* Typhimurium	P22-like phages (STMP1, STMP2 STMP3, STMP4, STMP5, STMP6, STMP7, STMP10, STMP11, STMP14, STMP15, STMP16, STMP17, STMP20, STMP21, STMP22, STMP23, STMP24, STMP26, STMP27, STMP28, STMP29, STMP32, STMP35)	Anderson typing-phage, *Podovirus*, *Lederbergvirus* short tail, lytic, narrow to moderate host range	[67,68]
		ES18-like phages (STMP8, ATMP18)	Anderson typing-phages, *Podovirus*, lambdoid long tailed, lytic	[67,68]
		SETP3-like phages (STMP12, ATMP13, SenTO17)	Anderson typing-phages, *Podovirus*, long noncontractile tailed, lytic	[67,68]
PT1, PT4, PT6, PT6a, PT7, PT8, PT11, PT13, PT13a, PT15, PT19, PT24, PT29, PT47; well known among the numerous phage types	*S.* Enteritidis	Phage 1	Ward typing phages, *Siphoviridae*-like, temperate, tailed, lysogenization, can convert PT4 to PT-1, turbid plaques	[69]
		Phage 2	Ward typing phages, *Siphoviridae*-like, temperate, tailed, lysogenization, convert PT4 to PT-1 or PT-8, turbid plaques	[69]
		Phage 3	Ward typing phages, *Siphoviridae*-like, temperate, tailed, lysogenization, convert PT4 to PT-8 or PT-3, turbid plaques	[69]
		Phage 6	Ward typing phages, *Siphoviridae*-like, temperate, tailed, lysogenization, convert PT6a to PT4, PT7, PT13 to PT13a, PT15 to PT11	[69]
		Phage phiPT1	*Siphoviridae*-like, lytic	[70]

## Data Availability

No new data were created or analyzed in this study.
